# A Weighted Genetic Risk Score of Adult Glioma Susceptibility Loci Associated with Pediatric Brain Tumor Risk

**DOI:** 10.1038/s41598-019-54701-1

**Published:** 2019-12-02

**Authors:** Maral Adel Fahmideh, Catharina Lavebratt, Giorgio Tettamanti, Joachim Schüz, Martin Röösli, Kristina Kjaerheim, Michael A. Grotzer, Christoffer Johansen, Claudia E. Kuehni, Birgitta Lannering, Lisbeth S. Schmidt, Hatef Darabi, Maria Feychting

**Affiliations:** 1 Department of Medicine, Section of Epidemiology and Population Sciences, Dan L. Duncan Comprehensive Cancer Center, Baylor College of Medicine, One Baylor Plaza, Houston, TX 77030 USA; 2 Unit of Epidemiology, Institute of Environmental Medicine, Karolinska Institutet, Nobels väg 13, SE-171 77 Stockholm, Sweden; 3 Neurogenetics Unit, Department of Molecular Medicine and Surgery, Karolinska Institutet, and Center for Molecular Medicine, Karolinska University Hospital, L8:00, SE-171 76 Stockholm, Sweden; 4 Section of Environment and Radiation, International Agency for Research on Cancer (IARC), 150 Cours Albert Thomas, 69372 Lyon CEDEX 08, Lyon, France; 5 Department of Epidemiology and Public Health, Swiss Tropical and Public Health Institute, Socinstrasse 57, 4002 Basel, Switzerland; 6 University of Basel, Petersplatz 1, 4003 Basel, Switzerland; 7 The Cancer Registry of Norway, Ullernchausseen 64, NO-0379 Oslo, Norway; 8 Department of Oncology, University Children’s Hospital of Zurich, Steinwiesstrasse 75, 8032 Zurich, Switzerland; 9 Unit of Survivorship, The Danish Cancer Society Research Centre, Strandboulevarden 49, DK-2100 Copenhagen, Denmark; 10 Oncology Department, Finsen Centre, 5073 Rigshospitalet, Blegdamsvej 9, DK-2100 Copenhagen, Denmark; 11 Swiss Childhood Cancer Registry, Institute of Social and Preventive Medicine, University of Bern, Mittelstrasse 43, 3012 Bern, Switzerland; 12 Children’s University Hospital of Bern, University of Bern, Freiburgstrasse 31, 3010 Bern, Switzerland; 13 Department of Pediatrics, University of Gothenburg, Smörslottsgatan 1, SE-416 85 Gothenburg, Sweden; 14 Department of Pediatrics, University Hospital Herlev, Herlev Ringvej 75, DK-2730 Copenhagen, Denmark; 15 Quantify, Hantverkargatan 8, SE-112 21 Stockholm, Sweden

**Keywords:** Cancer genetics, Cancer genetics

## Abstract

Genetic risk score (GRS) is used to demonstrate the genetic variants contributing to the polygenic architecture of complex diseases. By using a GRS, we have investigated the additive impact of the known adult glioma susceptibility loci on the pediatric brain tumor (PBT) risk and assessed the proportion of PBT heritability attributable to these susceptibility loci. A GRS was generated for PBTs based on the alleles and associated effect sizes derived from a previously published genome-wide association study on adult glioma. The GRS was calculated in CEFALO, a population-based case-control study of brain tumors in children and adolescents including saliva DNA of 245 cases and 489 controls. The unconditional logistic regression model was used to investigate the association between standardized GRS and risk of PBTs. To measure the variance explained by the effect of GRS, Nagelkerke pseudo-R^2^ was calculated. The GRS for adult brain tumors was associated with an increased risk of PBTs (OR 1.25 [95% CI 1.06–1.49], *p* = 0.009) and 0.3% of the variance in PBTs could be explained by the effect of GRS on the liability scale. This study provides evidence that heritable risks of PBTs are in-part attributable to some common genetic variants associated with adult glioma.

## Introduction

Genetic risk score (GRS), or polygenetic risk score, calculations are used to analyze the oligo- or polygenic architecture underlying complex genetic disorders. In such calculations, first, the alleles which are associated with a trait at a certain threshold and their effect sizes are identified in a discovery sample. Then, a GRS, being a composite score of the alleles identified in the discovery sample and weighted by the effect size (e.g. log odds ratio (OR)), is calculated for each individual in an independent target sample. Later, the regression model is applied to assess the association between the GRS and the phenotype in the target sample adjusting for covariates^1^.

The current study aimed to examine whether the genetic risk score for adult brain tumors, taking into account the combined effect of five susceptibility loci, is associated with risk of pediatric brain tumors and to assess the proportion of pediatric brain tumor heritability attributable to these known adult glioma susceptibility loci.

## Methods

We generated the GRS for pediatric brain tumors based on the alleles and associated effect sizes derived from a previously published genome-wide association study (GWAS) on adult glioma^2^, which in turn was based on the meta-analysis of six previously published GWAS and also two new GWAS including in total 12496 cases and 18190 controls. Quality control procedures and the GWAS results were described in details in the original paper^2^. The CEFALO study was employed as the target sample. CEFALO is a population-based case-control study of brain tumors in children and adolescents aged 7–19 years conducted in Sweden, Denmark, Norway, and Switzerland. Details of the study methods, single nucleotide polymorphism (SNP) selection, genotyping, and the quality control procedures have been described previously^3–5^. Briefly, saliva DNA of 245 cases and 489 controls was included in the study and was satisfactorily genotyped for 92 SNPs. The study was approved by the national data protection boards and ethical committees in all participating countries, all research was performed in accordance with relevant guidelines/regulations and written informed consent was obtained from all participants and/or their parents. Table 1 summarizes demographic characteristics of included cases and controls and the distributions of diagnostic pediatric brain tumor types. We selected the discovery sample for these analyses based on its similarity with the target sample for ethnicity, as well as its large sample size leading to the more accurate reported estimates.

**Table 1:** Characteristics of cases and controls.

Characteristics	Cases	Controls
No. of participants	245	489
**Sex**		
Males	136 (56%)	261 (53%)
Females	109 (44%)	228 (47%)
**Age-group (at reference date)**		
7–9 years old	48 (20%)	112 (23%)
10–14 years old	108 (44%)	219 (45%)
15–19 years old	89 (36%)	158 (32%)
**Country**		
Sweden	106 (43%)	174 (36%)
Norway	24 (10%)	62 (13%)
Denmark	62 (25%)	134 (27%)
Switzerland	53 (22%)	119(24%)
**Type of tumor (ICCC-3 group III)**^**a**^		
Astrocytoma (IIIb)	134 (55%)	
Other gliomas (IIId)	20 (8%)	
Ependymoma (IIIa)	19 (8%)	
Intracranial embryonal tumors (IIIc)	7 (3%)	
Other specified intracranial neoplasms (IIIe)	49 (20%)	
Unspecified intracranial neoplasm (IIIf)	16 (6%)	

^**a**^Restricted to ICD-O-3 location C71; patients with neurofibromatosis and tuberous sclerosis were excluded.

SNPs which were associated with the adult glioma risk at the genome-wide significance level in all the discovery, validation and combined results of either all glioma or non-glioblastoma glioma in the discovery sample were selected for the GRS analyses. Thus, the GRS in the CEFALO study was based on 5 SNPs (rs6010620, rs2736100, rs4977756, rs498872, rs2297440) and corresponding to the number of the risk alleles weighted by the logarithm of the ORs from the discovery sample across this set of SNPs (Table 2). PLINK was used to perform the analyses^6^.

**Table 2:** Summary results of the discovery sample for the SNPs included in genetic risk score analyses^2^.

SNP	Chr.	Gene	Location (bp)	Risk allele	Ref allele	OR	95% CI	P value
rs2736100	5	*TERT*	1339516	C	A	1.29	1.25–1.34	2.34 × 10^−45^
rs4977756	9	*CDKN2A/B*	22058652	G	A	1.28	1.23–1.32	1.46 × 10^−41^
rs498872	11	*PHLDB1*	117982577	A	G	1.14	1.10–1.18	4.09 × 10^−11^
rs6010620	20	*RTEL1*	61780283	G	A	1.34	1.29–1.40	2.81 × 10^−40^
rs297440	20	*RTEL1*	62312299	C	T	1.36	1.30–1.42	1.60 × 10^−42^

The GRS was subsequently standardized using its mean value and standard deviation (SD); for which the OR associated with it can then be interpreted as the increased (or decreased) odds of pediatric brain tumors for a one SD change in GRS. The unconditional logistic regression model was applied to evaluate whether the standardized GRS is associated with the risk of pediatric brain tumors adjusted for age, sex and country as covariates (full model); unmatched analyses have been performed since not all CEFALO participants provided saliva sample. To measure the variance explained by the effect of GRS, Nagelkerke pseudo-R^2^ was calculated as the difference of R^2^ in the full model compared to the reduced model including the covariates but not the GRS. The analyses were conducted using R^7^.

The study was approved by the Ethical Review Board in Stockholm, Sweden.

## Results

The results indicated that the standardized genetic risk score for adult brain tumors was associated with increased risk of pediatric brain tumors (OR 1.25 [95% CI 1.06–1.49], *p* = 0.009). The identified OR of 1.25 can be interpreted as: one SD increase in GRS is associated with a 25% increased odds of pediatric brain tumors. Moreover, 1.2% of the variance in pediatric brain tumors could be explained by the effect of the GRS (R^2^ = 0.012). The estimated disease prevalence used to transform the estimated heritability to the liability scale was 0.04%. The SNP-heritability on the liability scale was estimated to be 0.003.

The minimum detectable ORs for each individual SNPs rs6010620 (G), rs2736100 (C), rs4977756 (G), rs498872 (A), and rs2297440 (C) in this dataset of 245 cases and 489 controls were 1.61, 1.56, 1.56, 1.58, and 1.62, respectively.

## Discussion

This study was performed based on the hitherto largest series of pediatric brain tumor cases with the purpose to investigate the additive impact of the known adult glioma susceptibility loci on pediatric brain tumor risk and for the first time to investigate the similarity of SNP-heritability between adult and pediatric brain tumors.

To our knowledge, to date, two studies have assessed the heritability of adult glioma based on the known glioma susceptibility loci. Sampson *et al*. assessed the adult glioma heritability based on 10 loci (rs1412829, rs2157719, rs2736100, rs2853676, rs4295627, rs4809324, rs4977756, rs498872, rs6010620, rs891835) and reported the estimated SNP-heritability of 1.7% (*h*^2^ = 0.017)^8^. Moreover, Kinnersley *et al*. investigated the proportion of glioma heritability attributable to known glioma susceptibility loci (rs2736100, rs11979158, rs2252586, rs4295627, rs4977756, rs498872, rs6010620) and the estimated proportion of genetic variance of adult glioma explained by these seven risk loci was 1.6%^9^.

In the present study, we estimated that 0.3% of the variance in the pediatric brain tumors could be explained by the effect of the genetic risk score containing five known adult glioma susceptibility loci. This provides evidence that the heritable risks of pediatric brain tumors are in-part attributable to some common genetic variants associated with adult glioma. Also, previously, based on CEFALO study, we could show that adult and pediatric brain tumors have some genetic risk factors in common^4,5^. However, more polygenic analyses based on large sample sizes and big genotyping data for both adult and pediatric brain tumors are required to determine the similarity of genetic architecture in adult and pediatric brain tumors which is clinically relevant from a risk prediction and treatment perspective. In addition, this line of research provides the basis to develop comprehensive risk prediction models including both genetic and environmental factors for brain tumors that eventually may lead to brain tumor prediction and prevention. This can be achieved by incorporating large collaborative genetic association studies and high quality registration data and could be leveraged by refinement of molecular classification and development of somatic characteristics of brain tumors^10^.
